# 1110. Case Study. Enzymatic Debridement as a Blood-sparing Alternative: Pineapple Enzymes in the Burn Unit

**DOI:** 10.1093/jbcr/irag033.214

**Published:** 2026-04-06

**Authors:** Phil Fidler, Nevin George, Brice Bulotovich

**Affiliations:** Texas Tech University Health Sciences Center El Paso, El Paso, TX; Texas Tech University Health Sciences Center El Paso, El Paso, TX; Texas Tech University Health Sciences Center El Paso, El Paso, TX

## Abstract

**Patient Presentation (age range, injury details, relevant history):**

A 40-year-old female was involved in a vehicular collision in Mexico, sustaining an open right distal femoral fracture and 12% full-thickness burns to the back and left buttock. After initial workup in Mexico, she was transferred to the University Medical Center in El Paso for further care, where she was noted to be hypotensive and received whole blood; shortly after, she disclosed being a Jehovah’s Witness and declined further transfusions.

**Clinical Challenges:**

Her hemoglobin was initially stable after open reduction and internal fixation of the right femur, but it dropped to 7.0 g/dL by hospital day 3, prompting the burn team to pursue a blood-sparing strategy for debridement of large burn areas while minimizing blood loss due to her refusal of transfusion.

**Management Approach:**

On hospital day 6, she underwent partial thickness surgical debridement followed by application of the enzyme to 2550 cm^2^ of eschar on the back and buttock. Stoma paste was placed circumferentially around the burn wounds to confine the enzyme to regions of eschar. The enzyme was allowed to sit for five hours to allow for enzymatic processing, after which she was taken back to the OR for removal of necrotic tissue with a hydrosurgical system. A wound vac was then placed on the debrided tissue and total estimated blood loss across both procedures was approximately 10 mL.

**Outcomes:**

Total estimated blood loss across both procedures was approximately 10 mL. The combined approach allowed for effective eschar removal while minimizing blood loss.

**Lessons Learned:**

This case presents the successful use of enzymatic debridement as a blood-sparing adjunct in a Jehovah’s Witness patient with anemia and burns. Partial-thickness surgical excision combined with enzymatic debridement allowed for eschar removal while minimizing blood loss, underscoring the importance of individualizing burn care.

**Applicability to Practice:**

Partial-thickness surgical excision can be combined with enzymatic debridement for an effective means to achieve appropriate surgical debridement while minimizing blood loss.

